# Supplementary material for: Sevoflurane Dampens Acute Pulmonary Inflammation via the Adenosine Receptor A2B and Heme Oxygenase-1
Source: Cells. 2022 Mar 24;11(7):1094. doi: 10.3390/cells11071094 (PMC8997763; doi:10.3390/cells11071094)
Supplement: Supplementary file 1 [file cells-11-01094-s001.zip › cells-1627208-supplementary.pdf]

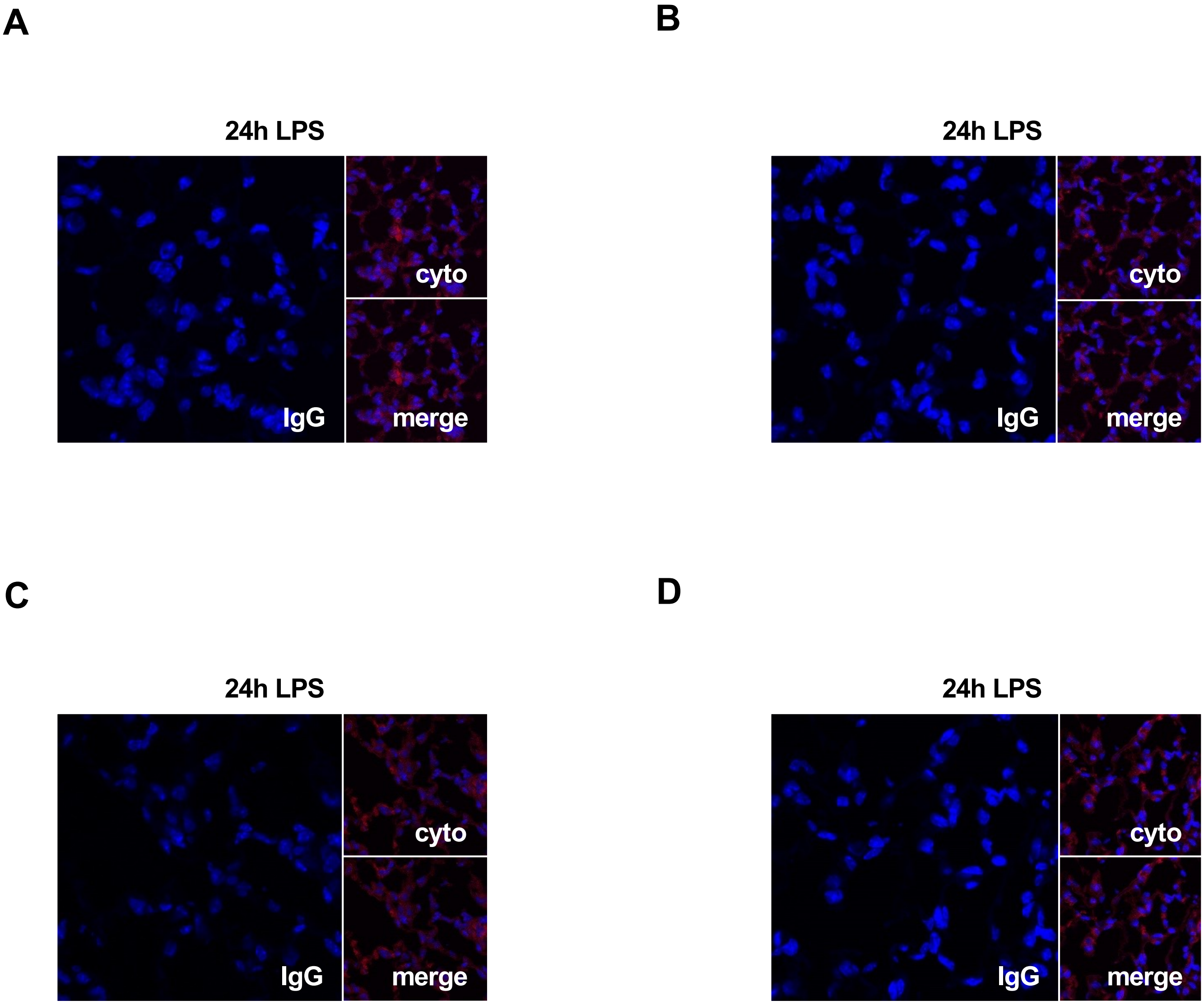

Supplementary Figure S1. (**A**) IgG control of zonula occludin-1 (ZO-1) and (**B**) occludin were performed by immunohistochemical staining from the lung tissue of wild-type animals (original magnification 63x; one representative image of four is shown; n=4). (**C**) Immunofluorescence experiments with unspecific IgG antibodies served as control staining for the detection heme oxygenase-1 (HO-1) and (**D**) adenosine A2B receptor (Adora2b) on the lung (original magnification 63x; one representative image of four is shown; n=4). DAPI was used as nuclear marker.

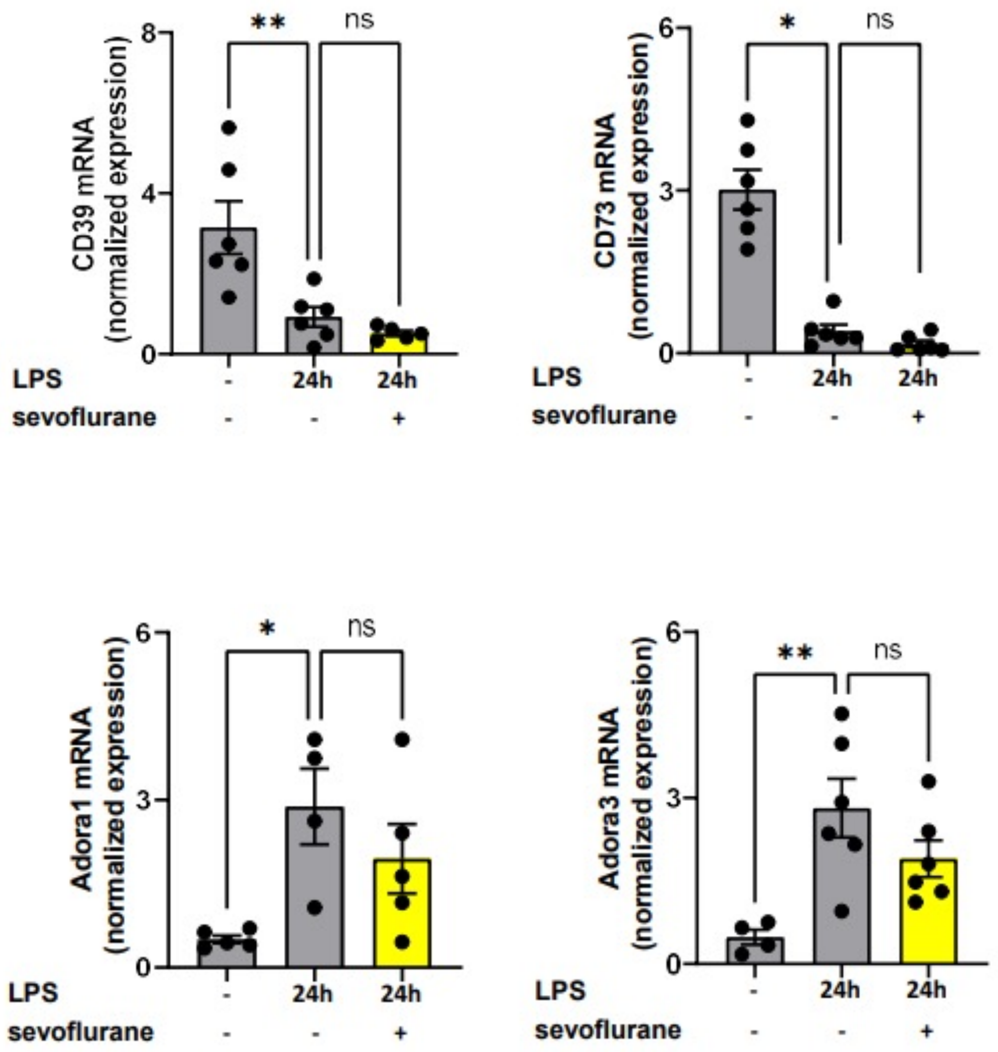

Supplementary Figure S2. Effects of sevoflurane von CD39, CD73, adenosine receptor A1 (Adora1), and Adora3 during acute pulmonary inflammation. CD39, CD73, Adora1, and Adora3 gene expression were evaluated in the pulmonary tissue 24 hours after lipopolysaccharide (LPS) stimulation by RT-PCR (n=4-6). Multiple group comparison was analyzed by one-way ANOVA and Bonferroni correction or Kruskal-Wallis test. Data are presented as mean ± SEM; ns = not significant; * P<0.05; ** P<0.01.

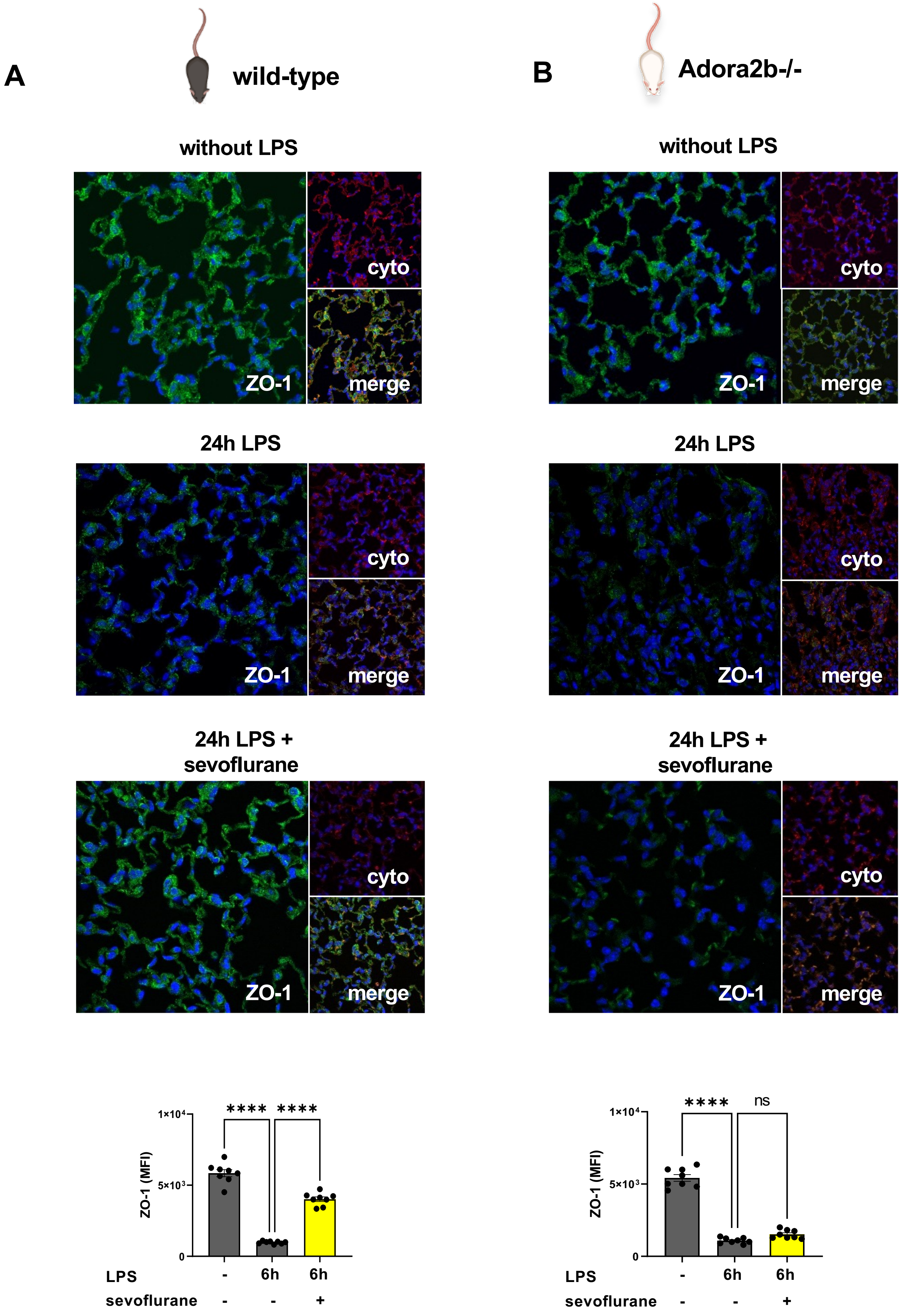

A
wild-type
B
Adora2b-/-
without LPS
cyto
ZO-1
merge
24h LPS
24h LPS +
sevoflurane
ZO-1 (MFI)
1×10⁴
5×10³
0
****
ns
LPS
-
6h
6h
sevoflurane
-
-
+

Supplementary Figure S3. (**A**) The lipopolysaccharide (LPS)-related expression of the tight junction protein zonula occludin-1 (ZO-1; green), in the lung tissue (red; cyto; cytokeratin) of wild-type and (**B**) adenosine receptor A2B (Adora2b) -/- mice were determined by immunofluorescence (original magnification x63; one representative image of three independent experiments is shown; n=3). DAPI (blue) was used as nuclear marker. Mean fluorescence intensities (MFI) of ZO-1 with or without LPS exposure were measured by using ImageJ (n=6-8). Statistical analyses were performed by one-way ANOVA + Bonferroni correction. Data are presented as mean ± SEM; ns = not significant; **** $P<0.0001$.

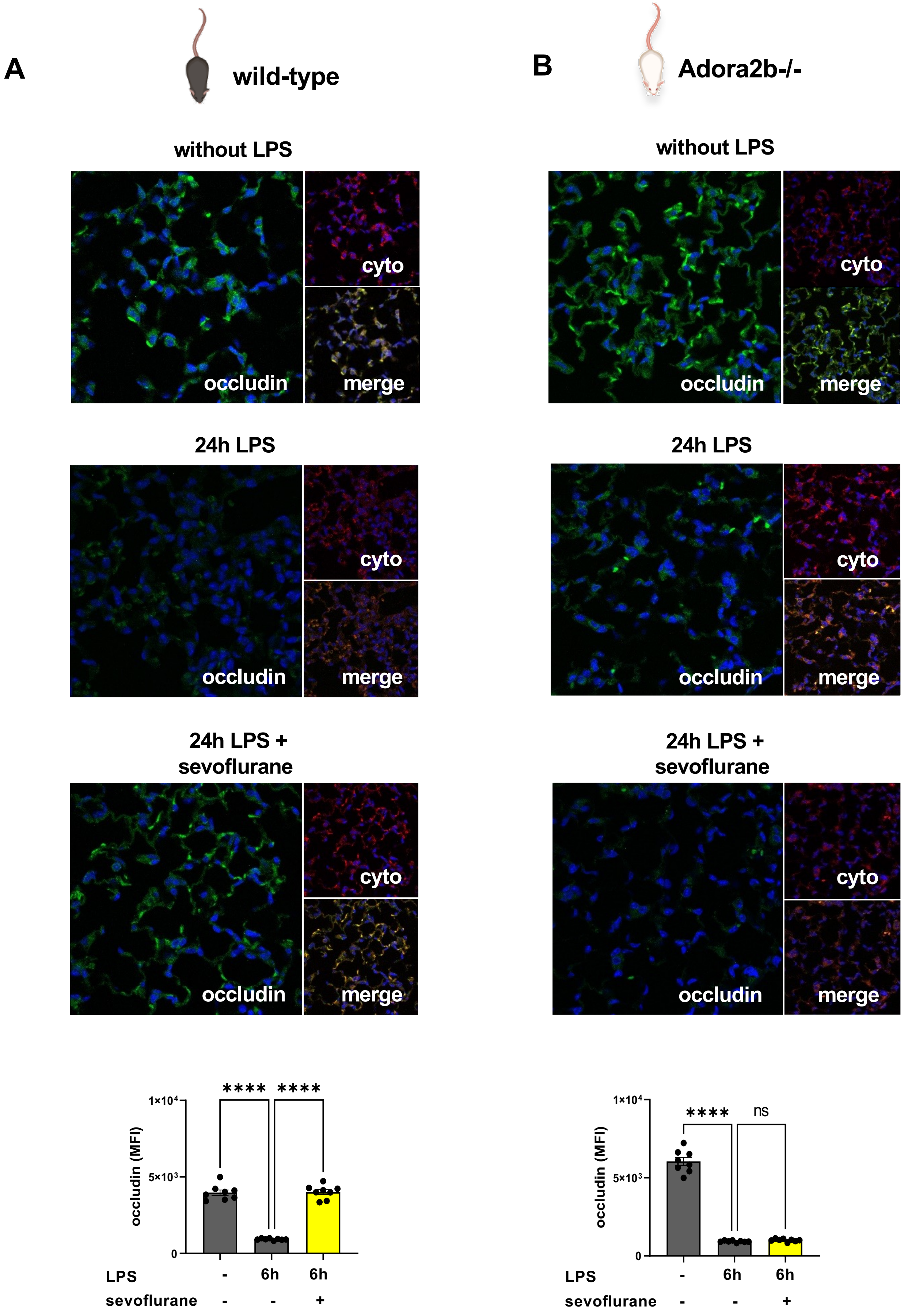
A
wild-type
without LPS
cyto
occludin
merge
24h LPS
cyto
occludin
merge
24h LPS +
sevoflurane
cyto
occludin
merge
1×10⁴
5×10³
0
occludin (MFI)
**** ****
LPS - 6h 6h
sevoflurane - - +
B
Adora2b-/-
without LPS
cyto
occludin
merge
24h LPS
cyto
occludin
merge
24h LPS +
sevoflurane
cyto
occludin
merge
1×10⁴
5×10³
0
occludin (MFI)
**** ns
LPS - 6h 6h
sevoflurane - - +

Supplementary Figure S4. (**A**) The lipopolysaccharide (LPS)-induced occludin (green) expression, in the lung tissue of wild-type and (**B**) adenosine receptor A2B (Adora2b) -/- mice were determined in the lung tissue (red; cyto; cytokeratin) by immunofluorescence (original magnification x63; one representative image of three independent experiments is shown; n=3). DAPI (blue) was used as nuclear marker. Mean fluorescence intensities (MFI) of occludin were measured at indicated conditions by using ImageJ (n=6-8). Statistical analyses were performed by one-way ANOVA + Bonferroni correction. Data are presented as mean ± SEM; ns = not significant; **** P<0.0001.

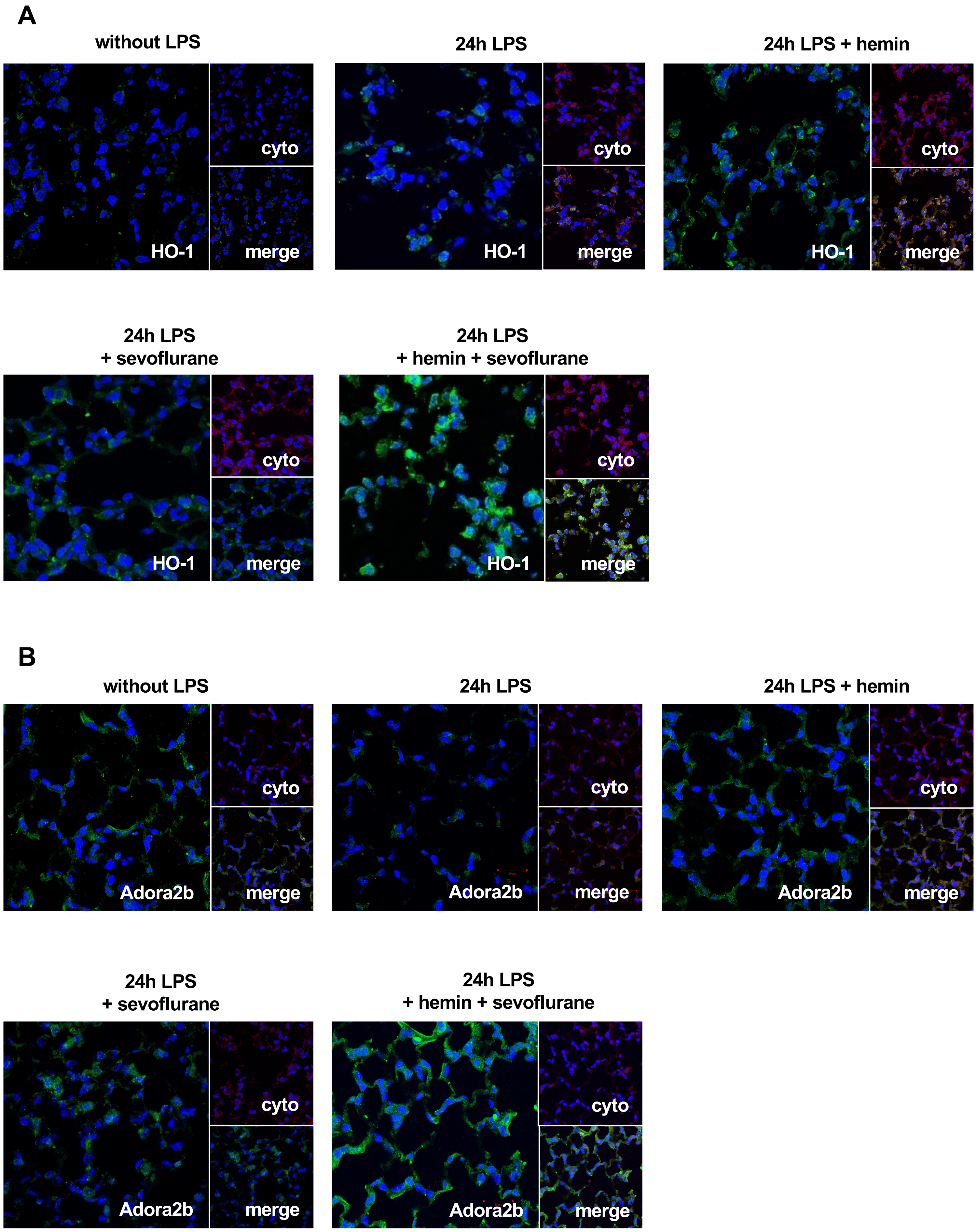
A
without LPS
HO-1
cyto
merge
24h LPS
HO-1
cyto
merge
24h LPS + hemin
HO-1
cyto
merge
24h LPS
+ sevoflurane
HO-1
cyto
merge
24h LPS
+ hemin + sevoflurane
HO-1
cyto
merge
B
without LPS
Adora2b
cyto
merge
24h LPS
Adora2b
cyto
merge
24h LPS + hemin
Adora2b
cyto
merge
24h LPS
+ sevoflurane
Adora2b
cyto
merge
24h LPS
+ hemin + sevoflurane
Adora2b
cyto
merge

Supplementary Figure S5. (**A**) Heme oxygenase-1 (HO-1) expression (green) in the pulmonary tissue (red; cyto; cytokeratin) was evaluated 24 hours after lipopolysaccharide (LPS) exposure by immunofluorescence at indicated conditions. (original magnification x63; one representative image of three independent experiments is shown; n=3). (**B**) Immunofluorescence staining of adenosine receptor A2B (Adora2b) surface expression (green) in lung tissue (red; cyto; cytokeratin) of wild-type animals were evaluated at indicated conditions (original magnification x63; one representative image of three independent experiments is shown; n=3). DAPI (blue) served as nuclear marker.
